# Lumbosacral stenosis in Labrador retriever military working dogs – an exomic exploratory study

**DOI:** 10.1186/s40575-017-0052-6

**Published:** 2017-10-23

**Authors:** Meenakshi Mukherjee, Jeryl C. Jones, Jianbo Yao

**Affiliations:** 1 Departments of Animal and Nutritional Sciences, Davis College of Agriculture, Natural Resources and Design, West Virginia University, Morgantown, WV 26506 USA; 2 Current address: 140 Poole Agricultural Center, Department of Animal and Veterinary Sciences, Clemson University, Clemson, 29634 USA

**Keywords:** Canine, Cauda equina syndrome, Computed tomography, CT, Military working dogs, Whole exome sequencing, Genetics, Genomics, Transthyretin, Lumbar spinal stenosis

## Abstract

**Background:**

Canine lumbosacral stenosis is defined as narrowing of the caudal lumbar and/or sacral vertebral canal. A risk factor for neurologic problems in many large sized breeds, lumbosacral stenosis can also cause early retirement in Labrador retriever military working dogs. Though vital for conservative management of the condition, early detection is complicated by the ambiguous nature of clinical signs of lumbosacral stenosis in stoic and high-drive Labrador retriever military working dogs. Though clinical diagnoses of lumbosacral stenosis using CT imaging are standard, they are usually not performed unless dogs present with clinical symptoms. Understanding the underlying genomic mechanisms would be beneficial in developing early detection methods for lumbosacral stenosis, which could prevent premature retirement in working dogs. The exomes of 8 young Labrador retriever military working dogs (4 affected and 4 unaffected by lumbosacral stenosis, phenotypically selected by CT image analyses from 40 dogs with no reported clinical signs of the condition) were sequenced to identify and annotate exonic variants between dogs negative and positive for lumbosacral stenosis.

**Results:**

Two-hundred and fifty-two variants were detected to be homozygous for the wild allele and either homozygous or heterozygous for the variant allele. Seventeen non-disruptive variants were detected that could affect protein effectiveness in 7 annotated (SCN1B, RGS9BP, ASXL3, TTR, LRRC16B, PTPRO, ZBBX) and 3 predicted genes (EEF1A1, DNAJA1, ZFX). No exonic variants were detected in any of the canine orthologues for human lumbar spinal stenosis candidate genes.

**Conclusions:**

TTR (transthyretin) gene could be a possible candidate for lumbosacral stenosis in Labrador retrievers based on previous human studies that have reported an association between human lumbar spinal stenosis and transthyretin protein amyloidosis. Other genes identified with exonic variants in this study but with no known published association with lumbosacral stenosis and/or lumbar spinal stenosis could also be candidate genes for future canine lumbosacral stenosis studies but their roles remain currently unknown. Human lumbar spinal stenosis candidate genes also cannot be ruled out as lumbosacral stenosis candidate genes. More definitive genetic investigations of this condition are needed before any genetic test for lumbosacral stenosis in Labrador retriever can be developed.

**Electronic supplementary material:**

The online version of this article (10.1186/s40575-017-0052-6) contains supplementary material, which is available to authorized users.

## Plain English summary

Labrador retrievers are popular for use as companion and working dogs worldwide. Like other large sized breeds; this breed is also prone to lower back diseases like lumbosacral stenosis. In this dog version of human lumbar spinal stenosis (leading cause of spinal surgery in Americans over 60), abnormal narrowing of the spinal canal in the lower back region causes several neurologic complications. Symptoms of lumbosacral stenosis are not always obvious and/or externally visible making detection difficult. If detected early, the condition is manageable by therapy, but by the time symptoms usually appear therapy becomes less effective. The problem is even more pronounced in working dogs that can get retired early – a major concern for military and police organizations that invest a lot of resources in recruiting and training working dogs. Expensive imaging methods such as computed tomography (CT) can confirm the diagnosis, but are not routinely performed without obvious symptoms. Diagnostic methods capable of detecting lumbosacral stenosis early are needed, preferably ones that are inexpensive, accurate and rapid (like blood/saliva tests). However it is important to understand the genetics of a disease to design such tests. Since we could not find any genetic studies of lumbosacral stenosis in Labrador retrievers, we explored said genetics in this preliminary study. We randomly selected 40 young Labrador retriever military working dogs with no outward signs of lumbosacral stenosis and assessed them for presence or absence of lumbosacral stenosis based on CT image analyses. We identified 4 dogs affected and 4 dogs unaffected by lumbosacral stenosis and sequenced their DNA to identify genetic differences between normal and diseased dogs. One mutation was identified in a gene that could be associated with lumbosacral stenosis – transthyretin (protein associated with human lumbar spinal stenosis). Other genes identified with mutations could also be important but we could not find evidence of this in the published literature, so more research is needed before they can be ruled out definitively. Once we can identify gene/s responsible for this condition, we will be that much closer to designing improved early detection methods for early detection of lumbosacral stenosis.

## Background

Working dogs are high-performance athletes that assist human team-members in supporting public service, national security and military missions in the U.S. and around the world [1]. Working dogs perform a variety of tasks such as sentry-and-patrol; search and rescue; mobility support for disabled persons; and detection of explosives, arson accelerants, and illegal drugs. Labrador retrievers are one of the most popular working dog breeds [2, 3]. As of 2016, the U.S. Military had an estimated 2300–2500 military working dogs (MWDs) across all branches of the Armed Forces [4, 5]. Each year the U.S. Military invests major financial and personnel resources in procuring, training and maintaining these MWDs in peak physical condition. According to a 2011 U.S. Government Pentagon memo, typical purchasing and training costs for a high quality MWD can go as high as $40,000 U.S. dollars [6]. Since 2001, the U.S. Department of Defense has spent more than $941,000 U.S. dollars towards this objective [4]. The demand for high quality working dogs (especially detection dogs) has been increasing in recent years [3]. But the demand often exceeds the supply [2], especially due to the high cost of breeding, raising and subsequent training of MWDs [3]. Ideally these trained working dogs are expected to maintain functionality for at least 10–12 years [7]. Early retirement of such trained MWDs means both a functional loss in the productivity of the team that depends on the trained dog as well as a financial loss for the Military.

Spinal diseases are a leading cause for early retirement in MWDs [8]. Lumbosacral stenosis (LS) is a common pathological condition affecting the canine lumbosacral spine, especially in large breeds like German shepherds and Labrador retrievers [9–18]. Military working dogs are also affected by LS [19], but there have been no breed-specific studies in Labrador retrievers.

Canine LS is defined as an abnormal narrowing of the lumbosacral canal, vertebral canal, and/or the intervertebral foramina [20]. This morphologic problem can be a risk factor for disability, often due to compression of the underlying neural and/or vascular tissues leading to clinical conditions like cauda equina syndrome (CES) [21]. Low back pain (LBP) on palpation of the lumbosacral spine is considered to be the primary clinical sign of LS [22]. However, there are drawbacks of using LBP status alone in diagnosis of LS in dogs: (i) symptoms can be intermittent with appearance only after hard physical exertion; (ii) symptoms can mimic those of other spinal diseases like intervertebral disc degeneration [23], degenerative sacroiliac joint disease [24], foraminal stenosis [25], and Schmorl’s nodes [26]; (iii) stoic dogs may not consistently vocalize pain; and (iv) dogs are specifically bred to have high-drive (desirable in MWDs, but can delay the detection of sub-clinical conditions like LS). A dog can be structurally LS positive, while remaining clinically asymptomatic until the condition worsens to such an extent that therapeutic and surgical options become unviable, and the only remaining course of action is retirement (in some severe cases, even euthanasia). Therefore improved methods for early detection of LS are critical for minimizing the risk of early retirement in these valuable canines.

Computed tomography (CT) is an established non-invasive diagnostic imaging technique for clinical diagnosis of canine LS [27, 28]. Morphological abnormalities within the canine lumbosacral canal that can lead to narrowing of the vertebral canal/intervertebral foramen (based on CT observations in surgically confirmed cases of LS) have been previously reported [17]. Based on these previously published CT diagnostic criteria, a recent study found the quantitative CT diagnosis of LS made by fat area ratio (FAR) measurements to be comparable with the qualitative CT diagnosis of LS made by a board-certified veterinary radiologist [29]. However, CT diagnoses of LS are not always possible especially since expensive imaging studies are not routinely performed unless a dog presents with some clinical signs of LS. This allows the condition to go undetected longer until the damage becomes irreversible and effectiveness of therapeutic interventions is significantly reduced. Previous studies have suggested LS therapy to be more effective in younger MWDs with mild clinical signs, with an increased chance of a successful return to active duty [19, 30]. Therefore, there is a need for methods capable of detecting LS early.

Similar to human lumbar spinal stenosis (LSS), canine LS has two distinct etiologies: congenital (idiopathic and developmental) or the more common acquired (degenerative and post-traumatic) [31]. Studies have reported association between genetic polymorphisms and degenerative LSS [32, 33]. Developmental LSS is usually observed in individuals with achondroplasia [34], and has known genetic causes [35]. Studies have also suggested that genetic factors could be influencing early manifestation of canine LS [36, 37]. This genetic predisposition of canine LS has been predominantly accounted for by lumbosacral transitional vertebra (LTV) – a congenital structural anomaly where the vertebra forms abnormally, usually between the last lumbar and first sacral vertebra [38, 39]. Presence of LTV can in turn cause CES due to abnormally narrow spinal canal (i.e. LS) [40]. An improved understanding of the currently unknown genomic mechanisms underlying LS in Labrador retrievers would be beneficial in designing improved diagnostic tests.

Whole exome sequencing (WES) method can explore the underlying genetic mechanisms of both Mendelian disorders and complex multi-factorial diseases [41–43]. Since coding regions constitute only about 1% of the whole genome, WES is an efficient, cost-effective and sensitive method for exploring the genomics of complex disorders (like cancer [44]; autism [45, 46]; hereditary myopathy in respiratory failure [47]; and osteogenesis imperfecta and Marfan’s syndrome [48]). Therefore WES approach was used for this initial genomic exploration study of the complex trait of LS in young Labrador retriever military working dogs without any outward signs of the condition. The exomes of a group of these dogs representing extremes of the LS phenotype (based on CT image analyses) were sequenced and annotated to identify possible exonic variants between LS negative and LS positive dogs. The findings from this exploratory study were intended to provide background data for future in-depth genetic association studies of LS in this vulnerable population of a predisposed breed.

## Methods

### Study subjects

This pilot study was prospective and exploratory by design. All procedures were approved by and conducted in accordance with requirements set by the institutional animal care and use committees of West Virginia University (protocol # 12–0409) and the Department of Defense Military Working Dog Veterinary Services (protocol # 2012–06; approval Aug. 12, 2012). Forty Labrador retriever military working dogs (MWDs) were prospectively selected from the MWD population housed at the Lackland Air Force Base (AFB) in San Antonio, TX. Inclusion criteria were as follows: available on the base during the time of data collection (from July 8, 2013 to July 13, 2013), ages between 1 and 5 years, approximately equal number of males and females, approximately equal number of yellow and black colored dogs, no outward signs of lumbosacral stenosis (visibly non-stenotic), and no health problems that would preclude sedation and CT scanning.

### Data collection

All data were collected with the help of personnel at the Holland Military Working Dog Hospital (Lackland AFB, San Antonio, TX). The demographic data collected included dog name and ID number, age, sex, breeder/vendor (if available) and dog duty status. A single licensed veterinarian performed all physical examinations and clinical data recording. Dog coat color was recorded along with and presence or absence of the following clinical signs: reaction to palpation of the LS junction, reaction to elevation of the tail, or reaction to extension of the hip joints. The dog’s handler and other technical staff were also interviewed to record approximate times the dog spent performing tasks such as jumping up onto or climbing over obstacles, or assuming an upright stance. Presence or absence of a history of reluctance to perform working tasks was also recorded. When available, the dog’s pedigree was also recorded.

Using a 3cm^3^ syringe and either a 22 or 20-gauge needle, a licensed veterinary technician on staff at the military dog hospital collected blood from the cephalic vein of each dog and placed the blood on commercially available sample collection cards (Whatman™ FTA™ cards, GE Healthcare UK Limited, Buckinghamshire, UK). After the cards were completely dry (as per the manufacturer’s instructions), they were inserted in specially designed and labeled protective pouches for uncontaminated (both bacterial and fungal) transport of the samples. The sample cards were then stored in airtight boxes at room temperature, in a dry location, and out of direct sunlight to prevent mold growth and degradation of the genetic material.

Immediately following physical examination and blood collection procedures, the same licensed veterinary technician sedated dogs using the hospital’s standard sedation protocols. A 32-slice CT scanner (Lightspeed, GE Medical Systems, Pewaukee, WI) present within the hospital premise was used to collect transverse scans of the lumbar and lumbosacral spine (L4 caudal to S1 cranial lumbar vertebrae). Technical settings used for all dogs were as follows: axial mode, 0.625 mm slice thickness, 120 kVp, 100 mA, body filter, and bone convolution kernel. All dogs were positioned in dorsal recumbency for CT scanning. Scans were acquired with the rear limbs first placed in a maximally extended position and then repeated with the rear limbs placed in a maximally flexed position. The same veterinary technician completed all positioning and scanning procedures under the supervision of a licensed veterinarian.

### Subject selection for exome sequencing

The selection of dogs for exome sequencing followed the extreme-phenotype sampling strategy [42, 49]. The selections were made based on qualitative and quantitative CT phenotyping at each of 8 vertebral locations, encompassing the cranial and caudal ends of 4 lumbar (L4, L5, L6 and L7) and 1 sacral (S1) vertebra – L4 caudal, L5 cranial, L5 caudal, L6 cranial, L6 caudal, L7 cranial, L7 caudal, and S1 cranial. Using previously published qualitative CT characteristics of surgically confirmed LS [17] and quantitative CT phenotyping techniques [29], a board-certified veterinary radiologist assigned all 40 dogs to one of two groups – unaffected or LS negative (no structural stenosis found at any of the 8 locations); and affected or LS positive (structural stenosis observed in at least in one of the 8 locations).

The previously published surgically-confirmed CT characteristics used by the board-certified veterinary radiologist to qualitatively phenotype LS at each location of each dog include the following: loss of epidural fat, increased soft tissue opacity, bulging of the intervertebral disc margin, vertebral endplate bone spurs, thecal sac displacement, focally narrowed vertebral canal, bulbous articular processes, articular process subluxation, and articular process osteophytes [17]. Qualitative assessments of LS based on these CT characteristics also had a strong correlation with quantitative CT variable of transverse-sectional canal fat area ratio (FAR) at 6 vertebral locations (L5 caudal, L6 cranial and caudal, L7 cranial and caudal, and S1 cranial) [29]. Therefore the FAR quantitative variable was calculated in the current study for the quantitative phenotyping of LS, but for 8 locations instead of previous 6 (two additional locations included L4 caudal and L5 cranial).

Eight dogs were then selected from these 40 dogs to represent the extremes of the LS phenotype – 4 LS negative and 4 LS positive. Dogs were assigned to the LS negative group when they had consistently high FAR values across all 8 vertebral locations and ideally a matching qualitative CT assessment of LS. Dogs were assigned to the LS positive group when they had the consistently low FAR values across all 8 vertebral locations and a matching qualitative CT assessment. Due to the subjective nature of qualitative LS diagnosis, preference for selection was given to the FAR values. Frequency of FAR values in all 8 locations was used for selection instead of the average of the 8 FAR values. This was done to reduce the “cancelling” effect that averaging FAR values could have when one location in a dog had high FAR, but another had low FAR. Clinical severity of LS at each location and for each dog was not graded in the current study.

### DNA isolation and exome sequencing

Genomic DNA of the 8 selected dogs (4 LS negative and 4 LS positive) was extracted from FTA cards using the GenSolve DNA Recovery Kit – GVR − 110 (GenTegra LLC, Pleasanton, CA), according to manufacturer’s protocol. An additional blood contamination purification step was done using the QIAamp Blood DNA Mini Kit (QIAGEN, Hilden, Germany). A commercially available exome capture kit (Illumina Nextera Rapid Exome Capture kit, Illumina Inc., San Diego, CA) was used for exome enrichment and capture, followed by rapid exome sequencing using the MiSeq system (Illumina Inc., San Diego, CA). Though the kit is designed to capture human exomes, there is enough sequence homology between human and dog genomes to ensure a successful capture of canine exomes with a large coverage area [50].

### Bioinformatics

The quality of the raw exome reads was analyzed using FastQC [51], and Trimmomatic [52] was used to filter out bad reads. The retention criteria were: leading bases with quality higher than 25, trailing not less than 20, four base sliding window cutoff of 25 and reads over 50 bases long. Each sample exome was then mapped to the reference dog genome CanFam3.1 (Broad CanFam3.1/canFam3 Assembly, September 2011) using Bowtie2 [53] with default parameters, followed by variant calling using SAMtools [54] and BCFtools [55]. Then SnpEff [56] was used to annotate each called variant, generating a variant call file (VCF). Another round of annotation was carried out to remove variants not called in all 8 samples, creating a second VCF file. A commercially available sequence annotation software package (Golden Helix SNP and Variation Suite, Golden Helix Inc., Bozeman, MT) was used to differentiate between the LS negative and LS positive dogs. Two separate selection settings were used to identify variants between the LS negative and LS positive groups. First setting: unaffected = “a/a”, affected = “b/b”. Second setting: unaffected = “a/a”, affected = “a/b” or “b/b” (where “a” = wild/reference allele; “b” = affected/alternate allele; “a/a” and “b/b” = homozygous for wild and alternate alleles, respectively; and “a/b” = heterozygous). The 2nd setting was intended to account for variant allele having an effect on LS in both homozygous and heterozygous states. Exonic variants were identified by aligning the variant list with the canine reference genome [57] CanFam3.1 (September, 2011 assembly release) [58] with the University of California Santa Cruz (UCSC) Dog Genome Browser [59, 60] The list of variants was annotated using Ensembl’s Variant Effect Predictor (VEP, Ensembl Gene annotation v83, December 2015) [61]. Since all canine genes have not been characterized, predicted genes for the genomic location were recorded (based on Ensembl predicted gene sets). The National Center for Biotechnology Information (NCBI) Basic Local Alignment Search Tool (BLAST) [62] was used to calculate percentage homology for the predicted canine gene sequences with the human and mouse reference gene sequences. The biological significance (i.e. association with clinical disorders) of the identified genes reported in either NCBI [63] or Ensembl [64] databases were also recorded.

A separate analysis was carried out to identify variants in previously reported human LSS candidate genes – genes associated with other closely related human musculoskeletal diseases like osteoarthritis (OA), Paget’s disease, degenerative disc disease (DDD), ossification of posterior longitudinal ligament (OPLL), osteogenesis imperfecta (OI) and Ehlers Danlos syndrome (EDS). The NCBI database was used to locate the canine orthologues of human LSS candidate genes. The genomic locations in the canine reference genome, along with the identification number (Gene ID) and validation statuses of these genes were also recorded. The variants matching the two sample-genotype parameters of the study (1st: LS negative = “a/a”, LS positive = “b/b”; 2nd: LS negative = “a/a”, LS positive = “a/b” or “b/b”) were then scanned to identify possible variants in identified canine orthologues. Variant annotation was also performed using the previously described methodology.

## Results

### Study subjects

The 40 sampled Labrador retrievers comprised of 20 males and 20 females. There were 24 black, 15 yellow, and 1 chocolate coated dogs. The average body weight of the study population was 28.48 kg (range 22.00–38.56 kg). The average age of the dogs in the study was 2.83 years. Sixteen of the 40 dogs were negative for signs of low back pain (LBP). The remaining 24 dogs displayed equivocal signs of pain during physical examination. The demographic and clinical data of the 40 study subjects are further described in Additional file 1: Table S1.

### Subject selection for exome sequencing

The detailed CT phenotyping results (both qualitative assessment of LS and quantitative measure of LS by FAR) at all 8 vertebral locations for all 40 dogs in the study are listed in Additional file 2: Table S2. Also listed was the total number of vertebral levels diagnosed as LS positive in each dog. The first 8 dogs selected for WES had to be excluded from further analyses due to insufficient purity of the extracted DNA. A second set of 8 dogs was then selected from the remaining 32 dogs using the same criteria. DNA samples of these 8 dogs (4 LS negative and 4 LS positive) were found to be of sufficient purity for further analyses and were selected for exome sequencing. These 8 dogs are highlighted in both Additional file 1: Table S1 and Additional file 2: Table S2 (^a^LS negative group, ^b^LS positive group). Additionally, the demographic and clinical data of the 8 dogs selected for exome sequencing are described with more detail in Table 1. All 4 LS negative dogs were females (ages 1–3), and 3 out of these 4 dogs were related to each other (one dam and two offsprings from the same litter). All 4 LS positive dogs were males between the ages of 3 to 5, and unrelated to each other. The LS negative dogs on average weighed less than the LS positive dogs. But no distinct difference was observed between the LS negative and LS positive with regards to low back pain status, coat color and/or work status of each dog.

**Table 1:** Demographic and clinical data of dogs selected for exome sequencing (*N* = 8)

Characteristic	CT LS negative	CT LS positive
Age (Average)	1, 1, 2, 3 (1.75)	3, 4, 4, 5 (4)
Sex	4 F	4 M
Mean weight	24.05	31.75
LBP status	3 E, 1 N	2 E, 2 N
Coat color	3 B, 1 Y	3 B, 1 Y
Work status	2 BR, 2 IT^a^	2 IT, 1 TA, 1 HH^b^

The demographic and clinical data of the 8 Labrador retriever military working dogs selected for exome sequencing representing LS negative and LS positive groups (based on qualitative CT diagnosis of lumbosacral stenosis by licensed veterinary radiologist)

^a^Denotes former breeders (BR) that were spayed and placed in training (IT)

^b^Denotes hospital hold (HH) for *T. cruzi* infection (medical condition unrelated to LS)

Legend: *CT* computed tomography, *LS* lumbosacral stenosis, *M* male, *F* female, *LBP* low back pain, *LBP status “E”* equivocal, *LBP status “N”* no, *B* black coat color, *Y* yellow coat color, *BR* breeder, *IT* In-Training, *TA* training aide, *HH* hospital hold

### Exome sequencing and bioinformatics

The sequencing runs for all 8 dogs (4 LS negative and 4 LS positive) resulted in fairly even representation among samples. After eliminating poor-quality raw exome reads, alignment with the reference canine genome (Broad CanFam3.1/canFam3 Assembly, September 2011) had a good alignment value (95 + %). A total of 110,980 variants were identified in all 8 samples, but 439 had to be excluded because they could not be assigned to any known canine chromosome number. Of the remaining 110,541 variants, there were no exonic variants that matched the 1st sample genotype setting: LS negative (“a/a”) vs. LS positive (“b/b”). There were 252 variants that matched the 2nd sample genotype setting: LS negative (“a/a”) vs. LS positive (either “a/b” or “b/b”). Manual perusal of these 252 variants identified 82 exonic variants spread out across a total of 33 genes, both annotated and predicted (Additional file 3: Table S3). The percentage homology of these 33 genes with their orthologous human and mouse genes are reported in Table 2. Functions and biological significance (associations with clinical syndromes) of these 33 genes as previously reported are listed in Additional file 4: Table S4 However, the functional impact of these 82 exonic variants could not be assessed within this manual method.

**Table 2:** Canine genes with exonic single nucleotide variants and percentage homology with human and mouse orthologues

	Dog (*Canis familiaris*)		Human (*Homo sapiens*)			Mouse (*Mus musculus*)		
No.	Chr. No.	Ensembl predicted gene name	Chr. No.	Sequence identity (%)	E-value	Chr. No.	Sequence identity (%)	E-value
1	1	DOCK8	9	93	2 e^-90	19	88	2 e^-70
2	1	SLC7A10	19	93	1 e^-74	7	91	1 e^-69
3	1	RGS9BP^a^	19	81	1 e^−167	7	76	4 e^-98
4	1	TSHZ3	19	91	0	7	87	0
5	4	KIAA1279 a.k.a. KIF1BP	10	88	0	10	81	0
6	6	TFR2	7	88	5 e^-52	5	87	4 e^-37
7	7	KIF21B	1	76	2 e^−173	1	91	7 e^-79
8	7	DTL	1	92	0	1	85	0
9	7	ASXL3^a^	18	87	0	18	81	0
10	7	TRAPPC8	18	90	0	18	81	0
11	7	TTR^a^	18	94	4 e^-24	18	NA	NA
12	8	LRRC16B^a^	14	90	3 e^−109	14	89	8 e^−109
13	8	ADCK1	14	93	1 e^-75	12	92	2 e^-74
14	10	ADD2 a.k.a. ADDB	2	84	3 e^−133	6	93	6 e^-55
15	11	SPOCK1	5	91	2 e^−139	13	90	3 e^-72
16	13	RNF139	8	92	0	15	91	0
17	13	TATDN1	8	86	1 e^-48	15	88	0
18	14	EEF1A1^b^	6	88	0	9	86	0
19	14	AGR2	7	85	2 e^-32	12	92	3 e^-19
20	14	DNAJA1^b^	9	89	0	4	89	0
21	14	TMEM168	7	81	0	6	88	0
22	15	CPE	4	82	0	9	88	3 e^-6
23	17	GREB1 or KIAA0575	2	86	0	12	79	3 e^-109
24	20	BSN or ZNF231	3	88	0	9	84	0
25	20	ABHD8	19	87	0	8	90	2 e^-53
26	21	FOLR2 or FBP	11	94	1 e^-73	7	81	2 e^-26
27	25	PALLD	4	84	0	8	80	3 e^-86
28	27	ABCC9	12	95	5 e^-100	6	86	2 e^-64
29	27	PTPRO^a^	12	78	0	6	91	2 e^-41
30	32	SMARCAD1	4	92	0	6	82	0
31	34	ZBBX^a^	3	84	2 e^-78	3	80	2 e^-42
32	X	ZFX^b^	X	91	0	X	85	0
33	X	USP9X	X	93	0	X	86	0

List of genes with exonic variants identified in the canine genome between the 4 LS negative and 4 LS positive Labrador retrievers in the exome sequencing study. Also listed are the percentage homologies between the canine genes (validated or predicted, according to NCBI database) and the human and mouse orthologues

^a^Denotes genes annotated by VEP as carrying moderate impact variants

^b^Denotes genes identified by VEP as carrying moderate impact variants but not annotated due to the uncharacterized nature of the canine gene

**Fig. 1:**
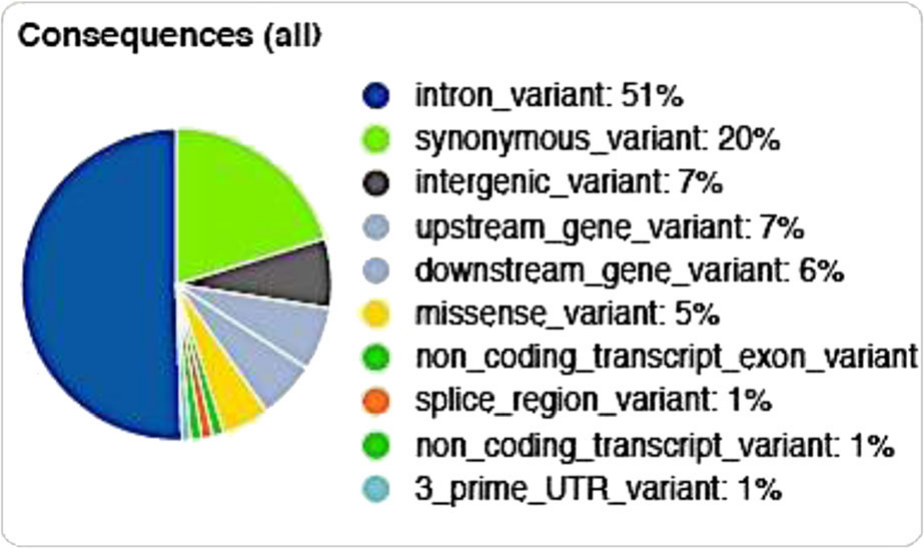
Consequences of the 229 variants analyzed by Ensembl’s Variant Effect Predictor (VEP)

**Fig. 2:**
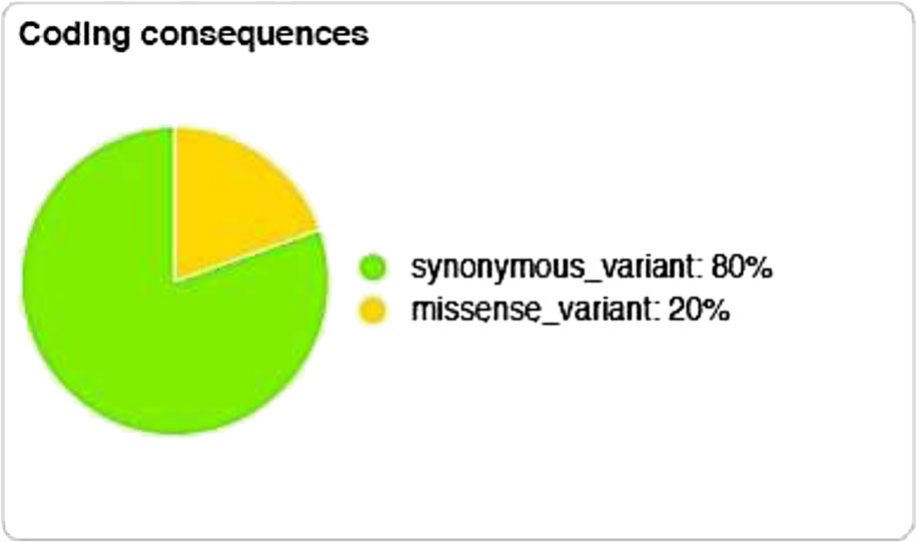
Percentage breakdown of exonic variants with coding regions based on type of mutation analyzed by Ensembl’s Variant Effect Predictor (VEP)

The 252 variants matching the 2nd sample genotype setting were also annotated using Ensembl’s VEP. Twenty-three of the 252 variants did not parse in the second method, so only 229 could be analyzed by VEP (165 of which were novel). All possible consequences of these 229 variants are summarized in Fig. 1. Majority of the variants detected (80%) were synonymous mutations, and the remaining (20%) were missense mutations (Fig. 2). Functional impacts of all 252 variants were assessed by VEP. No high impact variants (disruptive to protein function by causing protein truncation, loss-of-function and/or nonsense-mediated decay) could be identified. However, VEP was able to identify 17 moderate impact variants (non-disruptive but might affect the effectiveness of the protein) in 7 annotated (SCN1B, RGS9BP, ASXL3, TTR, LRRC16B, PTPRO, ZBBX) and 3 predicted genes (EEF1A1, DNAJA1, ZFX) (Table 3). Genomic locations were used to assign the un-annotated variants to predicted genes (NCBI). Seventy low impact variants (mostly harmless and/or unlikely to influence protein activity) were also identified spread across 24 genes. But no exonic variants were detected in any of the canine orthologues for the human LSS candidate genes (Table 4).

**Table 3:** Moderate impact exonic single nucleotide variants identified by Ensembl’s Variant Effect Predictor (VEP)

Location	Variant allele	Symbol	Gene	Exon	Amino acid	Codons	Strand
1:117,510,670	T	SCN1B	ENSCAFG00000007129	1/5	A/T	Gca/Aca	-1
1:119,322,126	T	RGS9BP	ENSCAFG00000007509	1/1	R/H	cGc/cAc	-1
7:56,104,802	T	ASXL3	ENSCAFG00000017980	10/11	T/N	aCt/aAt	-1
7:56,104,802	T	ASXL3	ENSCAFG00000017980	4/5	T/N	aCt/aAt	-1
7:57,946,958	C	TTR	ENSCAFG00000018046	1/4	S/G	Agc/Ggc	-1
8:4,065,167	A	LRRC16B	ENSCAFG00000011712	16/40	A/T	Gcc/Acc	1
14:26,883,912	A	–	ENSCAFG00000009915	4/8	A/T	Gcc/Acc	1
14:36,176,692	C	–	ENSCAFG00000009635	4/6	W/R	Tgg/Cgg	1
14:36,176,713	C	–	ENSCAFG00000009635	5/6	C/R	Tgt/Cgt	1
27:31,190,558	C	PTPRO	ENSCAFG00000012789	2/27	N/S	aAc/aGc	-1
34:32,184,727	C	ZBBX	ENSCAFG00000014517	18/20	K/R	aAa/aGa	-1
34:32,184,727	C	ZBBX	ENSCAFG00000014517	16/17	K/R	aAa/aGa	-1
X:19,788,059	A	–	ENSCAFG00000013408	6/7	T/N	aCc/aAc	1
X:19,789,132	T	–	ENSCAFG00000013408	7/7	T/S	Acc/Tcc	1
X:19,789,321	A	–	ENSCAFG00000013408	7/7	A/T	Gcc/Acc	1
X:19,789,651	G	–	ENSCAFG00000013408	7/7	L/V	Ctt/Gtt	1
X:19,789,906	T	–	ENSCAFG00000013408	7/7	L/F	Ctc/Ttc	1

List of moderate impact variants identified by VEP in the canine exome. Also listed are the position (chromosome number and loci), variant allele, gene name and annotation, and the amino acid change of the exonic variant. Variants without gene symbol represent canine genes that have not been characterized yet

**Table 4:** Human lumbar spinal stenosis candidate genes and location in canine genome

Human LSS Candidate genes	Human Musculo-skeletal diseases associated with Human LSS	*Homo sapiens* Chromosome number	*Canis familiaris* Chromosome number	Canine gene NCBI Gene ID	Canine Gene Validation Status
COL1A1	LSS	17	CF 9	403,651	Provisional
COL1A2	LSS, OI, EDS	7	CF 14	403,824	Provisional
COL2A1	OA, LSS	12	CF 27	403,826	Provisional
COL9A1	OA, LSS	6	CF 12	481,873	Model
COL9A2	OA, LSS, DDD	1	CF 15	607,609	Validated
COL9A3	OA, LSS, DDD	20	CF 24	612,430	Validated
COL11A1	OA, LSS	1	CF 6	100,685,969	Model
COL11A2	OA, LSS, DDD, OPLL	6	CF 12	481,734	Model
VDR	DDD, LSS	12	CF 27	486,588	Model
MMP-3	DDD, LSS	11	CF 5	403,445	Provisional

List of human LSS candidate genes, based on previous studies. Also listed are the human diseases associated with human LSS, human gene chromosome number, chromosome number of the canine orthologue, NCBI gene ID and validation status of the canine gene

Legend: CF *canis familiaris*, *LSS* lumbar spinal stenosis, *OA* osteoarthritis, *EDS* Ehlers-Danlos syndrome, *OPLL* ossification of posterior longitudinal ligament, *OI* osteogenesis imperfecta, *DDD* degenerative disc disease, *COL(x)A(y)* collagen type (x) alpha (y), *MMP*-3 matrix metallo-peptidase 3, *VDR* vitamin D receptor

## Discussion

To the authors’ best knowledge there are no previous studies that have investigated the genomics of lumbosacral stenosis (LS) in Labrador retrievers. The current study is the first attempt at understanding the genomic mechanisms underlying LS in the Labrador retriever breed with the search focused on the protein-coding exome. The genes identified here with exonic variants could serve as candidate genes for future in-depth genetic studies of canine LS in this breed. Preliminary findings suggest that one of these variants warrants further investigation as a possible genetic risk factor for lumbosacral stenosis in Labrador retriever MWDs – a missense mutation (Serine ➔ Glycine) in the TTR (transthyretin) gene.

Genome-wide association studies (GWAS) have been the prevalent approach to identify variants associated with complex traits [65, 66]. But GWAS can be a difficult approach for initial exploratory studies – they usually require large sample sizes [67], and usually are able to only detect the common small-effect variants [43, 68], instead of many rare variants with combined larger effects [69, 70]. Comparatively, whole exome sequencing (WES) studies are possible in smaller sample sizes, and are unbiased in selection of variants. Whole exome sequencing studies are also preferred due to the relative ease of availability and inexpensiveness of the commercially available exome-capture platforms [71]. Therefore WES approach was selected for the initial exploration of the genomics underlying LS in this population of MWDs of the Labrador retriever breed.

Sampling the extremes of quantitative traits (or unusual samples of qualitative traits) can further reduce the required sample size of WES studies without sacrificing statistical power [72, 73]. This sampling strategy usually involves 2 phases – initial broad discovery phase where extreme sampling in small sample size identifies significant variants/genes; followed by large scale replication/validation phase where the significant findings from the discovery phase are replicated for verification in larger sample sizes. Therefore the current study can be considered to be the initial “discovery phase” of the genomic exploration of LS in Labrador retriever military working dogs using the extreme-phenotype sampling strategy. The MWD population of this breed was ideal for sampling the “unusual” qualitative LS phenotype since the breed is at high-risk for LS and working dogs usually display an earlier than normal incidence of LS. An additional level of this sampling strategy was carried out in the study by selecting dogs with the “extremes” of the quantitative LS phenotype (based on FAR). Thus attempts were made to maximize the statistical power of the study.

A physically strenuous lifestyle (like that led by human athletes) has previously been associated with premature degeneration of the musculoskeletal system [74]. Degenerative lumbar spinal stenosis (DLSS) is a common spinal degenerative disease [75]. Even though there are studies that have reported reluctance to work as a probable clinical sign of LS in working dogs [9, 19, 30, 76], there is a lack of scientific evidence testing the contribution of working tasks themselves on the disease pathology of LS. We hypothesize that even though canine LS is most often associated with aging related degenerative changes, the physically strenuous life led by military working dogs might be a contributing factor for the early incidence of a degenerative condition like LS in young athletic dogs. Our study focused on the Labrador retriever breed alone, but we propose a similar mechanism could be at play with LS in young MWDs of other breeds as well. Gene-environment interaction could also have had an effect on the phenotypic expression of a complex trait like LS, which in turn could have resulted in misleading genotype-phenotype associations. To determine the true effect of genetics alone, the effect of external variables needed to be minimized [66]. This was a benefit of using a single breed to explore the genetics underlying LS since selecting dogs from multiple breeds could have introduced genetic variation unrelated to LS disease pathology. Military working dogs housed at the same military base share a highly uniform life (extremely similar diet, exercise/training regimen, climate, healthcare access etc.). Therefore the advantage of using MWDs as the study subjects was the removal of various external/environmental sources of variation that could have lead to incorrect genotype-phenotype association in LS.

Previous studies of canine LS have reported certain biases with regards to the relationship between qualitative LS status and multiple demographic factors such as age, gender and body weight [9, 77–79]. Lumbosacral stenosis usually affects older dogs more than younger ones (aging related degenerative LS is more common form of LS), males more than females (faster growth rate, larger body weight and more popular choice as working dogs), and heavier dogs more than dogs that weigh less (male dogs tend to be heavier than females, so the gender and body weight trends coincide with each other). The current study can be split into two parts: 1st part – comprising the entire study population (*N* = 40, 7 LS negative, 33 LS positive); and 2nd part – sub-study of the dogs chosen for exome sequencing (*N* = 8, 4 LS negative, 4 LS positive). In 1st part of the study, the association between demographic factors and LS status mostly agreed with previous reports except with the gender ratio. There were roughly equal number of males and females in both LS negative and LS positive groups. And though the affected dogs were on average older than unaffected dogs, the difference was very small. In comparison, the association between demographic factors and LS statuses of the dogs in second part of the study consistently agreed with findings in previous studies. The gender bias was obvious since all 4 LS negative dogs were females and all 4 LS positive dogs were males [9, 77–79]. Dog coat color displayed no obvious trends in either parts of the study. Even though there was a slight overrepresentation of black dogs in the LS positive group in 1st part, this trend was not observed in 2nd part (WES). The average age as well as the average body weight of the affected dogs was more than that of the unaffected dogs in both parts of the study (in agreement with previous reports). Similar to a previous CT phenotyping study of LS [29], no correlation could be detected between LS status and low back pain status. Small size of each group (in both parts of the study) prevented statistical testing of the associations between demographic factors and LS statuses of the dogs.

Three of the 4 LS negative dogs selected for exome sequencing were related to each other – a dam and 2 offsprings from the same litter. The familial relationship reduced the essential sample size of the LS negative or control group in the exome sequencing study from 4 to 2, i.e. the 3 genetically related dogs sharing the same bloodline representing “dog 1” and the unrelated dog as “dog 2”. This could be a possible source of bias in the results. However, variant selection criterion for LS negative dogs (homozygous for the “wild” allele i.e. “a/a”) should have ensured that the reduced sample size of the LS negative group did not affect the variant sequence identity. Genetically related study subjects are a common issue in genetic studies where sampling is done in genetically isolated populations [80]. Analyses factoring in this effect would have been desirable, but they were beyond the scope of the current study since complete pedigree information of all 40 dogs in the study was not available (due to procurement of dogs from multiple vendors).

Clinical severity of LS was not graded in the current study. The definition of clinical severity is subjective, and based on qualitative and clinical assessment it can vary. Therefore for the purposes of the current study, the dogs were just given a binary assignment for LS – negative or positive, at each of the 8 vertebral locations assessed. The subjective assessment of LS severity made by practicing veterinary radiologists remains the gold standard for clinical applications. However the fat area ratio (FAR) variable is a quantifiable measure of LS [29] that could potentially be used for quantitatively grading the severity of LS. This potential LS severity grading system using FAR could also be applied to future quantitative trait loci (QTL) mapping studies, but the novel grading system needs further development and validation before it can be implemented in research setting.

Out of 110,980 total variants called in all 8 samples, only 252 met the study selection criteria (LS negative: homozygous for wild allele; LS positive: heterozygous/homozygous for variant allele). Even though Ensembl’s VEP could not identify any genes with high impact variants, it was able to identify 10 genes with moderate impact variants, and 24 genes with low impact variants (total = 34 genes). The manual method identified 82 exonic variants in a total of 33 protein-coding genes. Thus VEP was able to identify variants in one additional gene than the manual method. Three exonic variants could have possible associations with LS in Labrador retrievers based on findings in previous studies – a moderate impact variant in Transthyretin (TTR); and two low impact variants, one each in Folate Receptor 2 (FOLR2) and Ubiquitin Specific Peptidase 9 X-linked (USP9X).

A missense mutation (preserved length but with different amino acid) was identified in the TTR gene (Serine ➔ Glycine). Transthyretin is one of at least 30 different types of amyloid proteins that can be deposited in the human body [81]. Recent studies have reported association between transthyretin-derived amyloidosis (ATTR) and human senile systemic amyloidosis (SSA) – a group of disorders involving the localized deposition of amyloid proteins in a variety of tissues [82]. A 2011 study found an association between SSA and human lumbar spinal stenosis (LSS) in the Japanese population [83]. The study tested ligamentum flavum specimens from 36 individuals with LSS. Amyloid protein deposits were found in 19 of the 36 samples, with 16 of those 19 testing positive for ATTR. Another study has reported an association between ATTR and LSS within the Swedish population [84]. This study found ATTR deposits in 25 out of 26 samples (resected bone fragments, pieces of ligament and other connective tissues from patients undergoing LSS surgery). Amyloid protein deposits in the brain has been well investigated for several years because of the role it plays in Alzheimer’s disease and Down’s syndrome [85]. Amyloid deposits are also found in connective tissues like ligaments, tendons and cartilages especially in the joints (knee, hip, vertebrae) of elderly individuals [86–88]; but the biological significance of this type of amyloidosis is unknown [84]. Previous reports suggest that similar to LSS, ATTR is an aging-related degenerative condition usually observed in individuals older than sixty, and more commonly in males than in females. The ATTR condition is usually also accompanied by cardiomyopathy and carpal tunnel syndrome. Therefore, studies indicate that TTR could be a possible candidate gene for human LSS; and that TTR could also be a possible candidate gene for canine LS in Labrador retrievers.

A low-impact variant found in FOLR2 could also be a possible candidate gene for both human LSS and canine LS in Labrador retrievers. This gene was previously detected in macrophages in the synovial fluid of patients suffering from osteoarthritis [89], a condition well documented to be present concurrently with both human LSS and canine LS. Another low-impact variant that could be associated with LS was found in USP9X gene – a X-linked gene that escapes X-inactivation in mammalian females [90], resulting in females having twice the dose of the gene product than males. The sex-specific trend of canine LS (males affected almost twice as much as females) could be due to LS being a X-linked condition, with USP9X gene product playing a protective role in LS disease pathology. However, it should be noted that both FOLR2 and USP9X variants were reported by VEP as low impact, i.e. most likely “harmless” to protein functionality. Therefore the associations of these genes with LS could be circumstantial only, and insignificant to LS disease pathology. An important finding in this study was the absence of any exonic variants in the human LSS candidate genes. The authors acknowledge that the sample size of this study was too low to completely rule out the involvement of human LSS candidate genes in canine LS. Another possible reason for not completely ruling out the role of human LSS candidate genes in canine LS is that these genes could be associated with degenerative canine LS instead of the (suspected) idiopathic canine LS observed in current study population. The authors suggest that the young Labrador retriever MWDs in this study could be affected by idiopathic LS instead of degenerative LS since they are too young for normal degenerative changes commonly associated with aging to set in.

A possible limitation of this study is that the findings might not be generalizable to other breeds of military (or other) working dogs, or even non-working dogs of the same breed (Labrador retrievers). Future genetic studies in varying canine populations (other breeds of MWDs, other working groups of Labradors and other breeds, non-working Labradors and other breeds) accounting for known covariates (like age, sex and diet) in their study designs need to be carried out to understand the true role of genetic factors on LS. Future studies investigating the role of genetically related individual dogs in presentation of LS and the etiology of LS presented within the young MWD population would be of interest to the military as well as other agencies that procure and/or breed working dogs. Therefore it would also be desirable to have future LS genetic studies in genetically related dogs that include and factor in complete pedigree information in their analyses. Future studies with large sample sizes investigating the association between LS and demographic factors such as age, gender and body weight also need to be carried out to identify any possible relationships. Sequencing studies remain more informative than traditional GWAS for study of complex traits. Whole genome sequencing (WGS) are even more informative than whole exome sequencing (WES) studies since WES only explores the exome and many non-protein coding regions have previously been implicated to be biologically significant in complex traits [91]. However until WGS becomes cheaper, WES remains the cost-effective option for exploratory sequencing association studies of complex traits. Even though extreme-phenotype sampling gives higher power compared to random sampling in smaller sample sizes, the strategy is limited by its inability of being generalizable to the general population [71]. Additionally if the complex trait is influenced by multiple loci, the power of detecting small-effects loci is decreased [92]. Therefore large scale WES studies would be desirable that can investigate the genetics of LS without the extreme-phenotype sampling strategy. Association of variants identified in sequencing studies with the complex trait of interest is not definitive, so follow-up replication studies (comparison with GWAS, targeted genotyping of candidate genes, large scale unbiased re-sequencing) in larger samples are needed to verify significance of the variants identified in the previous discovery phase [71]. But similar to discovery phase studies, replication studies also cannot signal causality. To understand the actual role of the variants on the disease mechanism, and/or to identify causation, further molecular/cellular experiments (like expression QTL and in-vitro protein assays) need to be carried out.

## Conclusions

In conclusion, lumbosacral stenosis is a major concern for the U.S. Military since it is a leading risk factor for early retirement in MWDs, especially due to delayed detection. Better diagnostic methods are needed that can detect LS early, so that therapeutic interventions can be effective. As current study findings suggest, LS is not limited to older dogs. Thereby suggesting the presence of idiopathic instead of degenerative etiology of LS in at least this population of dogs (young Labrador retriever MWDs). The current study is the first breed-specific genetic study of LS. The exomes of 8 young MWDs (4 LS negative and 4 LS positive) of this breed were investigated. No high-impact variants were identified, but 17 moderate and 70 low impact variants were. One moderate-impact variant was detected in a gene that could be a possible “candidate” gene for future in-depth genetic studies of LS since it codes for a protein that has previously been associated with human LSS – TTR (transthyretin). No exonic variants were detected in any of the previously reported human LSS candidate genes. However, the findings of the current study should be interpreted with caution and no genes should be ruled out yet. These preliminary findings are a result of an exploratory study that was based on a very specific population of dogs – young military working dogs of Labrador retriever breed. Further investigations are necessary to validate the findings of this study in larger samples of young Labrador retriever MWDs. Further studies are also needed to test whether the current study findings could be generalized to other breeds of dogs – both working as well as non-working dogs. Findings of this study are solely intended to provide a starting-point for future in-depth genetic studies of LS in dogs.

## Additional files

Additional file 1: Table S1. The demographic and clinical data of all 40 Labrador retriever military working dogs in the study. (XLSX 40 kb)

Additional file 2: Table S2. The qualitative and quantitative CT phenotyping of all 40 Labrador retriever military working dogs in the study. (XLSX 55 kb)

Additional file 3: Table S3. List of 82 exonic variants and the 33 genes they belong to, as identified in the manual method to match the sample genotype parameters. Also listed are the positions (chromosome number and loci) of the variants in the canine genome, the gene symbols they correspond to, and the sequence variation detected. Instances where the gene is read in reverse, parentheses were used to denote sequence of both strands (sense: within parentheses; reverse: outside parentheses). (XLSX 39 kb)

Additional file 4: Table S4. List of 33 genes identified with exonic variants and their biological significance (as identified from biological databases and previously published literature). (XLSX 48 kb)

## Abbreviations

CT: Computed tomography
FAR: Fat area ratio
LBP: Low back pain
LS: Lumbosacral stenosis
LSS: Lumbar spinal stenosis
MWD: Military working dog
SSA: Senile systemic amyloidosis
WES: Whole exome sequencing
